# Assessing the Function of the *ZFP90* Variant rs1170426 in SLE and the Association Between SLE Drug Target and Susceptibility Genes

**DOI:** 10.3389/fimmu.2021.611515

**Published:** 2021-03-16

**Authors:** Tingting Zhu, Yuandi Huang, Danfeng Qian, Yuming Sheng, Chaowen Zhang, Shirui Chen, Hui Zhang, Hui Wang, Xuejun Zhang, Junlin Liu, Changhai Ding, Lu Liu

**Affiliations:** ^1^Department of Dermatology, The First Affiliated Hospital, Anhui Medical University, Hefei, China; ^2^Department of Rheumatology and Immunology, Arthritis Research Institute, The First Affiliated Hospital of Anhui Medical University, Hefei, China; ^3^Department of Dermatology, Lu’an People’s Hospital, Lu’an, China; ^4^Department of Dermatology, The Second Affiliated Hospital, Hainan Medical University, Haikou, China; ^5^Clinical Research Centre, Zhujiang Hospital, Southern Medical University, Zhujiang, China; ^6^Menzies Institute for Medical Research, University of Tasmania, Hobart, TAS, Australia; ^7^Department of Medical and Molecular Genetics, King’s College London, London, United Kingdom

**Keywords:** *ZFP90*, single nucleotide polymorphism, systemic lupus erythematosus, eQTL, SLE drug target genes

## Abstract

A genome-wide association study (GWAS) has discovered that a polymorphism in the *ZFP90* gene is associated with systemic lupus erythematosus (SLE). In this study, we explored the candidate function of a *ZFP90* variant (rs1170426) in the context of SLE and detected the relationship between SLE susceptible genes and SLE drug target genes. First, we investigated the regulatory role of rs1170426 on *ZFP90* expression by expression quantitative trait loci (eQTL) analysis in peripheral blood mononuclear cells (PBMCs), T, B, and monocytes cells and annotated the regulatory function of rs1170426 using bioinformatic databases. Second, we compared the case-control difference in *ZFP90* expression levels. Third, we analyzed the association of genotype and *ZFP90* expression levels with SLE clinical characters. Last, we showed the interaction of SLE susceptibility genes with SLE drug target genes. Subjects with the risk allele “C” of rs1170426 had lower expression levels of *ZFP90* in PBMCs (*P* = 0.006) and CD8+ T cells (*P* = 0.003) from controls. SLE cases also had lower expression levels compared with controls (*P* = 2.78E-9). After correction for multiple testing, the *ZFP90* expression levels were related to serositis (FDR *p* = 0.004), arthritis (FDR *p* = 0.020), hematological involvement (FDR *p* = 0.021), and increased C-reactive protein (CRP) (FDR *p* = 0.005) in cases. Furthermore, the SLE susceptible genes and the recognized SLE drug target genes were more likely to act upon each other compared with non-SLE genetic genes (OR = 2.701, *P* = 1.80E-5). These findings suggest that *ZFP90* might play a role in the pathogenesis of SLE, and SLE genetics would contribute to therapeutic drug discovery.

## Introduction

Systemic lupus erythematosus (SLE) is a chronic multi-system autoimmune disease that mostly affects women of childbearing age (1). Though the pathogenesis of SLE is still unclear, inherited susceptibility is an important aspect (2). The risk allele “C” of rs1170426 was first identified to be significantly associated with SLE in Han Chinese and European populations (**Supplementary Table 1**) (3).

Zinc finger proteins (*ZFP*) are a diversified family of proteins performing numerous biological functions (4), such as regulating gene expression in many tissues (5). The expression of *ZFP90* is involved in various diseases, for example, colorectal cancer (6), cardiac dysfunction (7), intellectual disability (8), and obesity (9). In this study, we aimed to determine if rs1170426 was associated with SLE pathogenesis by affecting the expression of *ZFP90*. Previous studies have proved that the discoveries from GWAS play a vital role in pharmaceutical development and drug repositioning (10, 11). To assess the function of SLE genes in pharmaceutical development, we also aimed to map the protein-protein interaction (PPI) network between SLE susceptibility genes and recognized SLE drug target genes, using the drug database.

## Methods

### Subjects

To test the mRNA expression level of *ZFP90*, we recruited 135 SLE cases and 130 healthy controls. The demographic characteristics of the samples included in the analysis were summarized in **Table 1**. The genotyping and gene expression study of four immune cells was conducted in 116 other healthy controls. All subjects were Han Chinese and enrolled from the First Affiliated Hospital of Anhui Medical University, Hefei, Anhui Province, China. SLE cases were diagnosed by two or more consultants in the Rheumatology or Dermatology Department according to the revised American College of Rheumatology (ACR) SLE classification criteria (12). All clinical phenotypes of SLE cases were defined by the ACR SLE classification criteria. The clinically verified healthy controls had no history of SLE, family history of SLE, or any other autoimmune diseases. Each subject signed informed consent to participate in this study, which was performed following the 1964 Declaration of Helsinki and approved by the Ethical Committee of Anhui Medical University.

**Table 1:** Demographic characteristics of the study subjects.

Characteristic	SLE Cases n = 117	Healthy Controls n = 126	*P*
Gender			
Female	109	125	/
Male	8	1	/
Age (years)^1^	37.23 ± 11.86	36.34 ± 9.33	/
Medications			
Corticosteroids use	113 (96.6%)	/	/
Antimalarials use	74 (63.2%)	/	/
Immunosuppressive use	31 (26.5%)	/	/
Genotype			0.653
CC	4 (3.4%)	2 (1.6%)	/ / /
CT	34 (29.1%)	38 (30.2%)	
TT	79 (67.5%)	86 (68.3%)	
Allele			0.693
C	42 (17.9%)	42 (16.7%)	/ /
T	192 (82.1%)	210 (83.3%)	

^1^The age of the subjects is given at sample collection.

A P value is considered statistically significant if <0.05.

### Isolation of PBMCs, T Cells, B Cells, and Monocytes

The whole blood was collected from the above subjects using an anti-coagulation tube. We removed the plasma after centrifugal isolation (RT, 400 × g, 5 min) and added an equal volume of phosphate-buffered saline (PBS) to replace the removed plasma. The diluted blood was layered onto Ficoll-Hypaque Solution. The PBMCs were isolated by density-gradient centrifugation. The fresh PBMCs of 116 healthy controls were incubated for 15 min at 4°C with the following fluorescent conjugated monoclonal antibodies: anti-CD3-FITC, anti-CD14-PE, anti-CD19-APC, anti-CD4-PERCP-CY5.5, anti-CD8-PE-CY7. Stained cells were sorted on a BD FACSAria III. CD4+T, CD8+T, monocytes, and B cells were identified as CD4+/CD3+, CD8+/CD3+, CD14+/CD3−, and CD19+/CD3−, respectively. All stained cells were sorted to >98% purity.

### Total RNA Isolation and qPCR

We extracted total mRNA from PBMCs and immune cell subsets using TRIzol Reagent. After reverse transcription of RNA (400 ng of each sample, using the PrimeScript RT reagent Kit) isolated from the samples, we used qPCR assays to confirm the relative expression of *ZFP90* mRNA in collected PBMCs. The glyceraldehyde phosphate dehydrogenase (*GAPDH*) acted as the internal control. Below primers were employed in the qPCR: *ZFP90* primers: forward 5′- CGCCCCAGGAATCAGTGACA -3′ and reverse 5′- GCTATAGTTCTCCAGCATCACATCC-3′. The experiment was conducted using the ViiA 7 Real-Time PCR System. The level of *ZFP90* mRNA expression was computed using the 2^-ΔΔCt^ method (13).

### Genotyping

We extracted the genomic DNA using a Wizard Genomic DNA Purification Kit (QIAGEN, Germany). For 135 SLE cases and 130 healthy controls, we genotyped rs1170426 of each sample using the improved multiplex ligation detection reaction (iMLDR) technology. For 116 other healthy controls, we genotyped each sample using Sanger sequencing. The sequencing products were conducted on the ABI3730XL automatic sequencer (Applied Biosystems).

### Functional Annotation and Bioinformatic Insights

We used HaploRegv4.1 (http://archive.broadinstitute.org/mammals/haploreg/haploreg.php) (14) and rSNPBase (http://rsnp.psych.ac.cn/) (15) databases to annotate the functional elements containing rs1170426. We evaluated the effect of rs1170426 on *ZFP90* expression in SLE implicated tissues or organs by using the Genotype-Tissue Expression (GTEx) Analysis Release V8 (http://www.gtexportal.org/home/) (16). In addition, we downloaded the RNA-seq data of EBV-transformed lymphocytes from the website (http://ebv-b.helmholtz-muenchen.de/) to address the influence of EBV infection on ZFP90 expression (17). We used the WashU Epigenome Browser (https://epigenomegateway.wustl.edu/) (18) to discover the epigenomic regulatory potential of rs1170426.

### The Association Between SLE Susceptibility Genes and Drug Target Genes

We got the target genes of SLE therapeutic drugs by consulting the DrugBank database (https://www.drugbank.ca/) (19) and Therapeutic target database (http://db.idrblab.org/ttd/) (20). We extracted PPI information from InWeb (21) to evaluate the potential relationship between SLE risk genes and drug target genes. Furthermore, we obtained the PPI network of *ZFP90* with drug target genes by using the BioGRID database (https://thebiogrid.org/) (22).

### Statistical Analysis

We used one-way analysis of variance to evaluate the cis-eQTL regulatory effect of rs1170426 on *ZFP90* and compared *ZFP90* mRNA expression levels in four kinds of immune cells. Mann-Whitney U test was used to detect the case-control difference in *ZFP90* mRNA expression level, the correlation between *ZFP90* mRNA expression level and clinical characteristics, the effect of treatment on *ZFP90* expression, as well as the difference in *ZFP90* mRNA expression level between cases and controls in each rs1170426 genotype group, which did not conform to the normal distribution. Chi-square test was applied to conduct association analysis in our cohort, genotype-phenotype analysis, and to analyze the difference of interaction probability between SLE risk genes and non-risk genes with drug targets. The PPI network diagram was plotted by Cytoscape software (version v3.8.0). SPSS (version 26.0) software and GraphPad Prism (version 8.01) were used for other data analysis and plotting. For multiple testing, a false discovery rate (FDR) p-value of 0.05 was used. The P-value (2 tails) of less than 0.05 indicated statistical significance.

## Results

### Association of rs1170426 With *ZFP90* mRNA Expression

**Figure 1:**
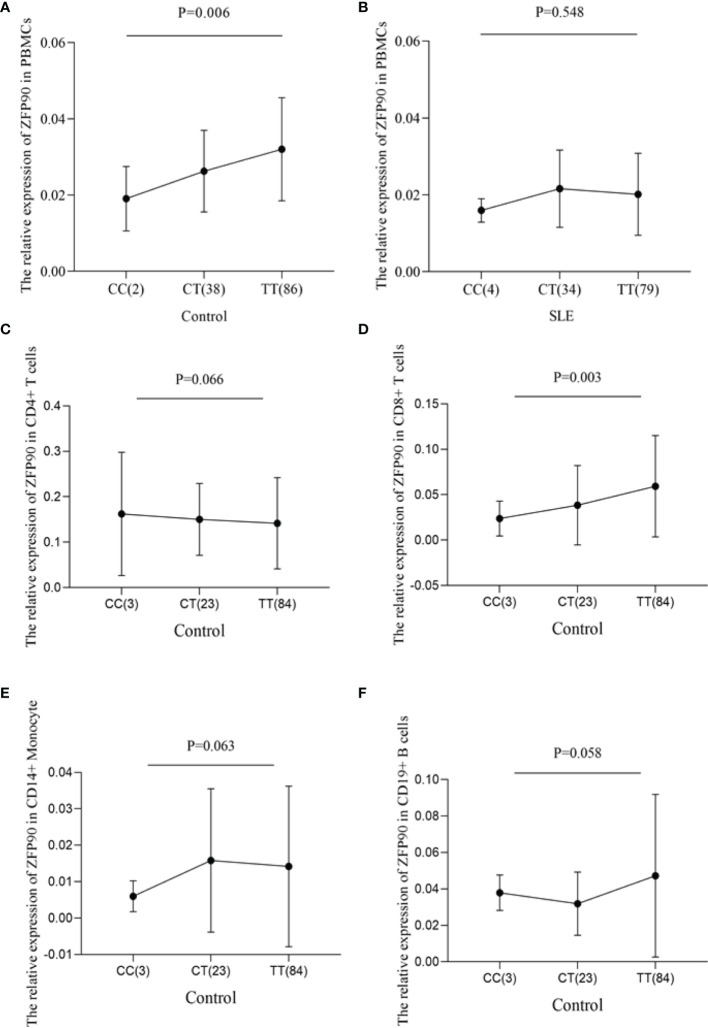
**(A)** The effect of rs1170426 on *ZFP90* mRNA expression levels in PBMCs from healthy controls. Of the 126 controls, 2 individuals with CC, 38 with CT, and 86 with TT were analyzed. The group with “CC” homozygous has the lowest expression levels (*P* = 0.006). **(B)** The effect of rs1170426 on *ZFP90* mRNA expression levels in PBMCs from SLE cases. Of the 117 cases, 4 individuals with CC, 34 with CT, and 79 with TT were analyzed. The expression did not significantly correlate with genotype of rs1170426 (*P* = 0.548). **(C–F)** The effect of rs1170426 on *ZFP90* mRNA expression levels in CD4+ T cells, CD8+ T cells, CD19+ B cells, and CD14+ monocytes from other 110 healthy controls. Of the 110 controls, 3 individuals with CC, 23 with CT, and 84 with TT were analyzed. *ZFP90* expression levels of samples with risk allele “C” of rs1170426 were significantly decreased in CD8+ T cells (*P* = 0.003).

To predict the regulatory function of rs1170426 on *ZFP90* mRNA expression, we performed eQTL analysis in PBMCs, CD4+ T, CD8+ T, CD14+ monocytes, and CD19+ B cells. As shown in **Figure 1A**, the risk allele “C” of SNP rs1170426 was correlated with lower expression levels of *ZFP90* in PBMCs extracted from healthy controls (*P* = 0.006). However, there was no significant association in SLE cases (*P* = 0.548) (**Figure 1B**). Considering the medicine would affect eQTL, we grouped the patients by medicine type (corticosteroids, antimalarials, and immunosuppressive) and conducted eQTL analysis in each group. The results are all negative (**Supplementary Table 2**). In the four kinds of immune cells, we only found rs1170426 was an eQTL for *ZFP90* in CD8+ T cells (*P* = 0.003) (**Figures 1C–F**).

### Detection of rs1170426 Regulatory Effects

**Figure 2:**
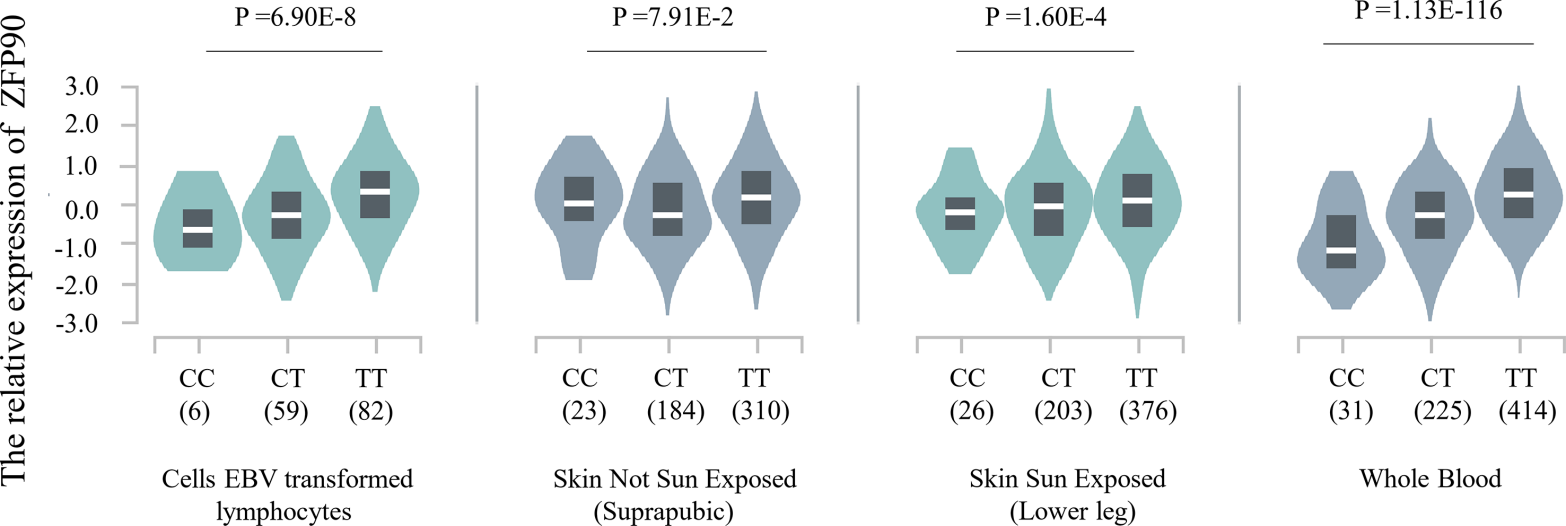
Plot showing the results of cis-eQTL analysis for SNP rs1170426 in EBV-transformed cells, sun-exposed skin, not sun-exposed skin, and whole blood (*P* = 6.90E-8, *P* = 7.91E-2, *P* = 1.60E-4, *P* = 1.13E-116, respectively).

**Figure 3:**
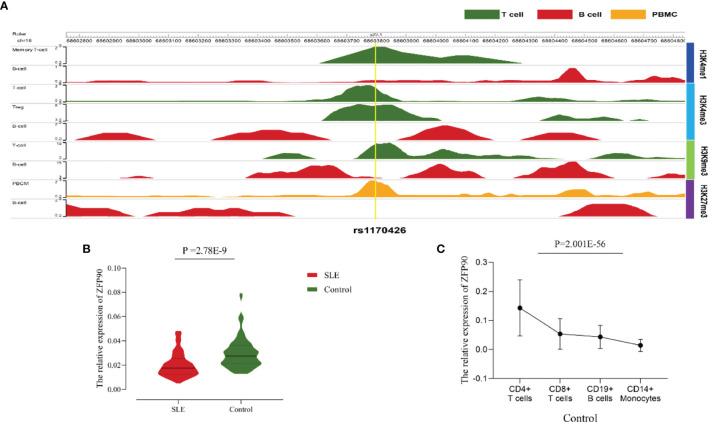
**(A)**The epigenome annotation results of rs1170426 in T cells (green), B cells (red), and PBMCs (orange). The signal can be found in T cells and PBMCs, while not in B cells. **(B)** Compared the *ZFP90* mRNA expression levels between SLE cases (n = 117, red) and healthy controls (n = 126, green) in PBMCs. The expression levels were lower in cases than in healthy controls (*P* =2.78E-9). **(C)** The *ZFP90* mRNA expression levels in four kinds of immune cells were remarkably different (*P* = 2.001E-56) and were higher in T cells.

The HaploReg v4.1 database showed rs1170426 located in the intron of *ZFP90* and overlapped with the binding site motifs of the transcription factor CCCTC-Binding Factor (CTCF) and Estrogen Receptor in T cells and monocytes (**Table 2**). It also played the role of a regulatory element in T cells and monocytes, i.e., gene enhancers marked by histone modifications histone H3 lysine 4 trimethylation (H3K4me3). We further found 158 SNPs in strong linkage disequilibrium (r^2^ > 0.8) with rs1170426 in the Asian population. Among them, 135 SNPs were regarded as regulatory SNPs (rSNPs) from SNPBase database. According to the GTEx Portal (version v8), rs1170426 is a cis-eQTL impacting on the expression of *ZFP90* in EBV-transformed cells, sun-exposed skin, not sun-exposed skin, and whole blood (*P* = 6.90E-8, *P* = 7.91E-2, *P* = 1.60E-4, *P* = 1.13E-116, respectively) (**Figure 2**). By analyzing the processed RNA-seq data of EBV transfected lymphocytes in controls, we found that EBV transformation does not affect *ZFP90* expression (**Supplementary Figure 1**). The epigenome annotation results show that the variant rs1170426 is located in the enhancer region of T cells and PBMCs respectively marked by H3 lysine 4 monomethylation (H3K4me1), H3K4me3, H3 lysine 9 trimethylation (H3K9me3), and H3 lysine 27 trimethylation (H3K27me3). However, we did not observe the signal in B cells (**Figure 3A**).

**Table 2:** Functional annotation of rs1170426.

SNP	Chr	Gene	Risk allele	Position (hg19)	Function	Enhancer histone marks	LD-proxy of rSNP (r^2^ > 0.8)^1^	Motifs
rs1170426	16q22.1	*ZFP90*	C	68603798	intron	T cell, Monocyte	Yes	Esr2, CTCF_disc1, CTCF_disc4, ERalpha-a_disc1

^1^SNP in strong LD (r^2^ > 0.8) with rSNPs;

rSNP, rSNPBase identified regulatory SNPs.

### Difference in *ZFP90* mRNA Expression Levels

A total of 135 cases and 130 controls were collected for qPCR, 117 SLE cases and 126 healthy controls passed quality control for inclusion in the subsequent analysis. The median level of *ZFP90* mRNA expression in SLE cases [Q_50_ = 0.018 (Q_25_ = 0.013, Q_75_ = 0.027)] was 1.5 times lower than in healthy controls [0.027 (0.021,0.036)]. Accordingly, the mRNA expression level of *ZFP90* was substantially decreased in SLE cases compared to healthy controls (*P* = 2.78E-9) (**Figure 3B**). Then, we divided the samples into three groups by rs1170426 genotype and compared the case-control *ZFP90* mRNA expression levels in each group, respectively. The results showed that the *ZFP90* mRNA expression levels of the “CT” and “TT” groups were significantly decreased in cases (*P* = 0.004 and 1.836E-9, respectively). However, there is no statistical significance in the “CC” group (*P* = 0.800) (**Supplementary Figure 2**). In addition, we compared the *ZFP90* expression level between patients with and without treatment by a certain class of drug (corticosteroids, antimalarials, and immunosuppressive). The results were not statistically significant (**Supplementary Table 2**).

At last, we found the *ZFP90* mRNA expression levels was remarkably different among four kinds of immune cells (*P* = 2.001E-56), including CD4+ T, CD8+ T, CD14+ monocytes, and CD19+ B cells. Compared to other two types of cells, it especially expressed higher in T cells (**Figure 3C**).

### The Correlation Between *ZFP90* mRNA Expression and Clinical Characteristics

**Figure 4:**
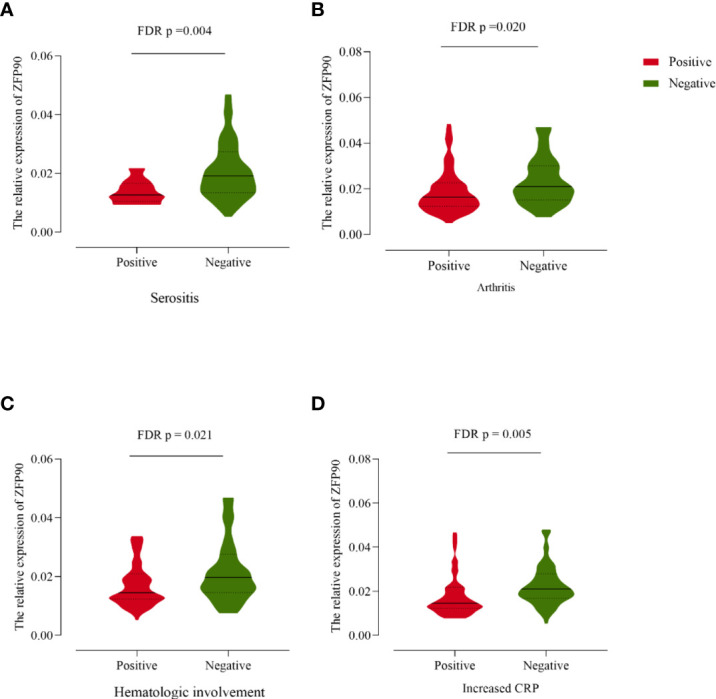
**(A)** The *ZFP90* mRNA expression levels were lower (FDR *p* = 0.004) in cases with serositis than those without. **(B)** The levels were lower (FDR *p* = 0.020) in cases with arthritis than those without. **(C)** The levels were lower (FDR *p* = 0.021) in cases with hematologic involvement than those without. **(D)** The levels were lower (FDR *p* = 0.005) in cases with increased CRP than those without.

Compared with SLE cases without serositis, arthritis, or hematological involvement, cases with these symptoms had much lower levels of *ZFP90* mRNA expression (FDR *p* = 0.004, FDR *p* = 0.020, FDR *p* = 0.021, respectively, **Figure 4**, **Table 3**). Moreover, the expression level was also significantly lower in cases with increased CRP than cases without a change in CRP (FDR *p* = 0.005).

**Table 3:** Association between *ZFP90* mRNA expression levels and SLE clinical characteristics in cases.

Clinical characteristics	Presence		Absence		FDR*_P_*^2^
	N^1^	Mean ± SD/Q_50_ (Q_25_,Q_75_)	N	Mean ± SD/Q_50_ (Q_25_,Q_75_)	
SLEDAI^3^ ≥10	63	0.019 (0.013,0.030)	54	0.017 (0.013,0.022)	0.132
Clinical manifestations					
Renal damage	77	0.019 (0.013, 0.026)	40	0.016 (0.012,0.025)	0.148
Malar rash	40	0.019 (0.013, 0.023)	77	0.018 (0.013, 0.023)	0.199
Discoid rash	14	0.018 (0.009,0.015)	103	0.018 (0.012,0.028)	0.199
Photosensitivity	10	0.024 (0.012,0.033)	107	0.017 (0.012,0.025)	0.165
Vasculitis	11	0.019 ± 0.010	106	0.018 (0.013,0.027)	0.191
Hematologic involvement	52	0.016 (0.012,0.025)	65	0.019 (0.015,0.028)	0.021*
Arthritis	42	0.016 (0.012,0.024)	75	0.021 (0.015,0.031)	0.020*
Mucosal ulcers	14	0.016 (0.016,0.028)	103	0.018 (0.013,0.028)	0.074
Central nervous system involvement	8	0.017 ± 0.006	109	0.018 (0.013,0.027)	0.177
Serositis	20	0.013 (0.011,0.018)	97	0.019 (0.013,0.029)	0.004*
Cardiac involvement	17	0.015 (0.012,0.022)	100	0.018 (0.013,0.028)	0.137
Laboratory indicators					
ANA (+)	114	0.017 (0.013,0.026)	3	0.065 ± 0.076	0.150
Anti-dsDNA (+)	66	0.018 (0.013,0.028)	51	0.017 (0.013,0.027)	0.206
Anti-SM (+)	59	0.018 (0.013,0.024)	58	0.018 (0.013,0.030)	0.200
Anti-RNP (+)	45	0.018 (0.014,0.027)	72	0.018 (0.013,0.028)	0.197
Increased CRP	66	0.015 (0.013,0.022)	51	0.021 (0.017,0.031)	0.005*
Increased ESR	90	0.017 (0.013,0.029)	27	0.018 (0.013,0.026)	0.200
Low complement	77	0.018 (0.013,0.028)	40	0.017 (0.013,0.024)	0.191

^1^The number of patients;

^2^ False Discovery Rate P value;

^3^Systemic Lupus Erythematosus Disease Activity Index;

*A FDR_P_ value is considered statistically significant if <0.05.

### Association Study and Genotype-Phenotype Analysis of rs1170426 and SLE

We found the frequency of the risk allele “C” in SLE cases was higher than in healthy controls, however, there is no statistical significance (**Table 1**). Then, we performed the genotype-phenotype analysis in two steps. First, we analyzed the correlation between genotype frequency and clinical character. Second, we conducted it for allele frequency. We only discovered that rs1170426 was statistically significant with arthritis involved (*P* = 0.037) (**Table 4**).

**Table 4:** The results of genotype-phenotype analysis.

Clinical characteristics	Genotype			*P^1^*	Allele		*P*
	CC (4)	CT (34)	TT (79)		C (42)	T (192)
SLEDAI^2^≥10	2^3^	17	44	0.907	21	105	0.661
Clinical manifestations							
Renal damage	3	22	52	0.927	28	126	0.775
Malar rash	2	8	30	0.294	12	68	0.439
Discoid rash	0	4	10	0.590	4	24	0.608
Photosensitivity	1	1	8	0.238	3	17	0.738
Vasculitis	0	3	8	0.657	3	19	0.591
Hematologic involvement	1	16	35	0.650	18	86	0.896
Arthritis	1	18	23	0.037*	20	64	0.065
Mucosal ulcers	1	6	7	0.307	8	20	0.107
Central nervous system involvement	0	2	6	0.716	2	14	0.558
Serositis	1	5	14	0.843	7	33	0.975
Cardiac involvement	1	6	10	0.649	8	26	0.332
Laboratory indicators							
ANA (+)	4	32	78	0.772	40	188	0.711
Anti-dsDNA (+)	3	20	43	0.622	26	106	0.353
Anti-SM (+)	3	16	40	0.590	22	96	0.639
Anti-RNP (+)	2	14	29	0.764	18	72	0.459
Increased CRP	3	14	49	0.122	20	112	0.247
Increased ESR	4	24	62	0.287	32	148	0.938
Low complement	2	23	52	0.730	27	127	0.937

^1^If number <5, choose Fisher’s Exact Test, otherwise Person Chi-Square;

^2^Systemic Lupus Erythematosus Disease Activity Index;

^3^The number of patients with both the clinical phenotype and a certain genotype or allele; *A P value is considered statistically significant if <0.05.

*A P value is considered statistically significant if <0.05.

### The PPI Information for SLE Susceptibility Genes and Drug Target Genes

**Figure 5:**
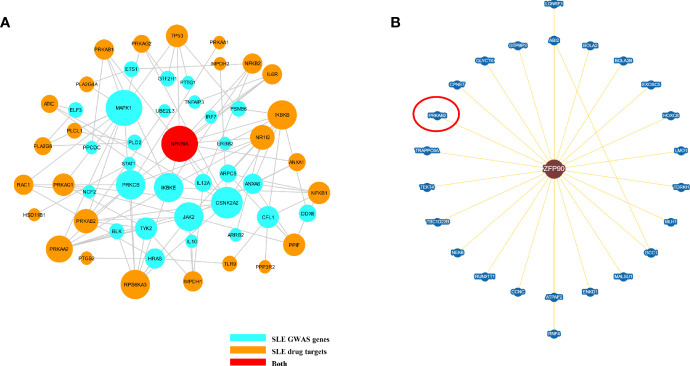
**(A)** The plot shows the PPI network of putative SLE risk genes and SLE drug target genes. SLE risk genes are shown as light blue nodes and SLE drugs target genes are shown as orange nodes. *NFKBIA*, which was shown as red node, is both SLE risk genes and SLE drug target genes. **(B)** This figure plots the protein interaction between *ZFP90* and drug-targeted genes. *PRKAB2* is a SLE drug-targeted gene (circled in red).

We summarized 86 published and putative SLE risk genes that achieved genome-wide significance (*P* < 5.0E-8) (23, 24) (**Supplementary Table 3**). We then discovered 41 SLE drug target genes by the DrugBank database and Therapeutic target database (**Supplementary Table 4**). From the Inweb database, 72 SLE susceptibility genes (without *ZFP90*) were found to have PPI information with other genes, of which 29 were found to have protein interactions with SLE drug-targeted genes (**Figure 5A**). However, only 2,496 out of 12,491 SLE unassociated genes had PPI information with SLE drug-targeted genes in the Inweb database. Although only *NFKBIA* was mapped as both SLE risk genes and SLE drug target genes, the probability of SLE drug-targeted genes interaction with SLE susceptibility genes was much higher than unassociated genes (OR = 2.701, *P* = 1.80E-5) (**Figure 5A**). In the BioGRID database, we further discovered protein interaction between *ZFP90* and *PRKAB2*, a drug target gene of SLE (**Figure 5B**).

## Discussion

SLE is a chronic autoimmune disease that often involves multiple organs, and its pathogenesis is related to numerous signaling pathways. GWAS is an efficient strategy to define disease susceptibility variants (25, 26). Our team has detected that rs1170426 located in the *ZFP90* region is strongly linked to SLE by a trans-ancestral GWAS meta-analysis (3). Nonetheless, its potential causal effect and clinical relevance remain to be investigated in SLE. In this study, we found rs1170426 was a cis-eQTL for *ZFP90* in PBMCs and primary CD8+T cells extracted from healthy controls. SLE cases expressed markedly lower transcriptional levels of *ZFP90* compared with controls. After correction for multiple testing, the *ZFP90* expression levels were still correlated with some clinical characters in cases. Importantly, we mapped the protein-protein interaction (PPI) network between SLE susceptibility genes and recognized SLE drug target genes.

*ZFP90* is a zinc-finger protein that contains 13 zinc finger domains and a KRAB domain (27). The dysregulation of *ZFP90* was associated with some autoimmune diseases, including inflammatory bowel disease, vitiligo, and multiple sclerosis (3). According to the expression data from GTEx, *ZFP90* is widely expressed in various tissues such as skin and whole blood. We also confirmed it abundantly expressed in PBMCs and especially in T cells. Through functional annotation using multiple databases, we revealed that the variant rs1170426 had a regulatory function on *ZFP90*. The SNP rs1170426 of *ZFP90* is predicted to span the binding site motifs of the transcription factor CTCF and Estrogen Receptor in T cells and monocytes. We also discovered rs1170426 was a cis-eQTL for *ZFP90* in healthy PBMCs and primary CD8+T cells. The CTCF has been reported to take part in regulating the aberrant gene expression in SLE T cells (28). The estrogen contributes to immune cell trafficking and inflammation (29), which advances the understanding of significant gender bias in SLE cases. These findings provide essential clues to explore the relationship between SLE and the variant rs1170426 of *ZFP90*.

*ZFP90* has been reported to regulate the activation of Ca^2+^ channels to a certain extent (7). An increased and prolonged Ca^2+^ influx was also observed in T cells of SLE patients (30). Altered Ca^2+^ flux can cause mitochondrial dysfunction and predispose to necrotic cell death (31). Increased cell necrosis may trigger the pro-inflammatory status resulting from the production of pro-inflammatory cytokines [such as TNF-α, interleukin-6 (IL-6) and IL-8] and interferon (32).

We also found *ZFP90* mRNA expression level was strongly linked with clinical characteristics. It was significantly lowered in SLE cases with serositis than those without. Serositis is one of the manifestations of lupus, which is often accompanied by the presence of infections. The exact pathogenesis of SLE serositis is still elusive. A study showed abnormal Ca^2+^ influx could lead to an inflammatory response associated with the onset of serositis (33). Our results also displayed that *ZFP90* mRNA expression level was lower in cases with arthritis than without. A previous study has shown that arthritis affects no less than 90% of SLE cases at some point during the course of the disease (34). It has been reported that IL-6, related to *ZFP90*, can promote arthritis and joint deformation in SLE cases (35). Consequently, we can reasonably suspect that the low expression of *ZFP90* promotes the production of pro-inflammatory factors leading to the onset of arthritis. Besides, some studies have shown that pro-inflammatory factors can stimulate the production of CRP (36, 37). Hence, in this study, SLE cases with a lower *ZFP90* mRNA expression level had an elevated CRP level. As we all know, infection is a common and leading cause of morbidity and mortality in SLE patients (38). CRP is generally associated with infection. So we would imagine the lower expression of *ZFP90* was associated with SLE through the infection pathway.

Hematopoietic stem cells (HSCs), which take on the potency to self-renew and differentiate to all lineage blood cells, play an essential role in hematopoiesis (39, 40). Studies have shown that *ZFP90* can be used as a transcription factor to participate in the regulation of HSC self-renewal and repopulation potential *in vivo* (41). Besides, excessive activation of the Wnt/β-catenin signaling pathway, an important signal pathway to participate in SLE (42), facilitates HSCs to lose the abilities of differentiation (43, 44). SLE cases with low expression of *ZFP90* are more likely to have a hematologic abnormality, perhaps due to the loss of the balance between proliferation and differentiation in HSCs.

In the PPI network of *ZFP90* and SLE drug target genes, *ZFP90* interacts with *PRKAB2. PRKAB2* can activate AMP-activated protein kinase (AMPK) in response to Ca^2+^ increase (45). *ZFP90* also participates in the regulation of Ca^2+^ channels and affects the flow of Ca^2+^ as mentioned above. This may contribute to explain the relationship between *ZFP90* and *PRKAB2*. *PRKAB2* is also one of the target genes of Fostamatinib that is a drug approved by the US Food and Drug Administration (FDA) in 2018 for the treatment of idiopathic thrombocytopenic purpura. Fostamatinib has been confirmed to suppress the development of lupus skin and kidney disease in lupus-prone mice (46). This find might promote that Fostamatinib would be used for SLE patients in the future. Choosing genetically supported targets can significantly increase the success rate of clinical drug development (11), which is supported by our analysis of the relationship between SLE susceptibility genes and drug target genes.

The current study still has some caveats. The limited sample size may weak the statistical power. Besides, as the real target could be mapped far away from the associated variants (47), lists of the causal genes for disease associations can be incomplete, and inaccurate at times.

In summary, we have dissected the possible pathogenic role of *ZFP90* in SLE and its impact on related clinical characteristics. Given the clinical characteristics, bioinformatics, and functional significance of genetic variation and the defined functional target gene, *ZFP90*, we draw the conclusion that the risk SNP locus of rs1170426 and its associated pathways might be meaningful for SLE, and targeting this pathway may be vital in the prevention or treatment of SLE. In addition, we provide evidence that the SLE genetics play important role in therapeutic drug discovery. However, further studies will be needed to elucidate this further.

## Data Availability Statement

The raw data supporting the conclusions of this article will be made available by the authors, without undue reservation.

## Author Contributions

LL conceptualized and supervised this study. CD, JL, and XZ contributed to the study design. TZ conducted data analysis and wrote the manuscript. LL polished and revised the manuscript. TZ and YH performed functional annotation and qPCR experiments. YH and DQ sorted the cells and extracted PBMC, DNA, and RNA. YS performed genotyping. CZ and SC conducted sample recruitment. HZ and HW collected clinical information. All authors contributed to the article and approved the submitted version.

## Funding

This study was supported financially by First batch of construction fund for innovation Capacity “135” Project (4601011226), Innovation research Group recipient cultivation Fund (4601011223), and Young Program (82003328) of National Natural Science Foundation of China (NSFC).

## Conflict of Interest

The authors declare that the research was conducted in the absence of any commercial or financial relationships that could be construed as a potential conflict of interest.

The reviewer YS declared a shared affiliation with several of the authors, TZ, YH, YS, CZ, SC, HZ, HW, XZ, CD, and LL, to the handling editor at the time of review.

The handling editor declared a shared affiliation, though no other collaboration, with one of the authors LL.
