# Corrigendum: Assessing the Function of the *ZFP90* Variant rs1170426 in SLE and the Association Between SLE Drug Target and Susceptibility Genes

**DOI:** 10.3389/fimmu.2022.840847

**Published:** 2022-02-18

**Authors:** Tingting Zhu, Yuandi Huang, Danfeng Qian, Yuming Sheng, Chaowen Zhang, Shirui Chen, Hui Zhang, Hui Wang, Xuejun Zhang, Junlin Liu, Changhai Ding, Lu Liu

**Affiliations:** ^1^ Department of Dermatology, The First Affiliated Hospital, Anhui Medical University, Hefei, China; ^2^ Department of Rheumatology and Immunology, Arthritis Research Institute, The First Affiliated Hospital of Anhui Medical University, Hefei, China; ^3^ Department of Dermatology, Lu’an People’s Hospital, Lu’an, China; ^4^ Department of Dermatology, The Second Affiliated Hospital, Hainan Medical University, Haikou, China; ^5^ Clinical Research Centre, Zhujiang Hospital, Southern Medical University, Zhujiang, China; ^6^ Menzies Institute for Medical Research, University of Tasmania, Hobart, TAS, Australia; ^7^ Department of Medical and Molecular Genetics, King’s College London, London, United Kingdom

**Keywords:** *ZFP90*, single nucleotide polymorphism, systemic lupus erythematosus, eQTL, SLE drug target genes

In the original article, there were mistakes in **Figures 1**, **3**, **4** as published. The number of CC genotype is not 10 but 2 in the legend of **Figure 1A**. The SNP ID is not rs2297550 but rs1170426 and the number of CT genotype is not 33 but 34 in the legend of **Figure 1B**. The number of CT genotype is not 33 but 34 in the label of **Figure 1B**. In **Figure 3**, the legends of **Figures 3B**, **3C** were inverted. In **Figure 4B**, the label of arthritis is not FDR *p* = 0.004 but FDR *p* = 0.020 as shown in **Table 3**. The corrected **Figures 1**, **3**, **4** appear below.

**Figure 1:**
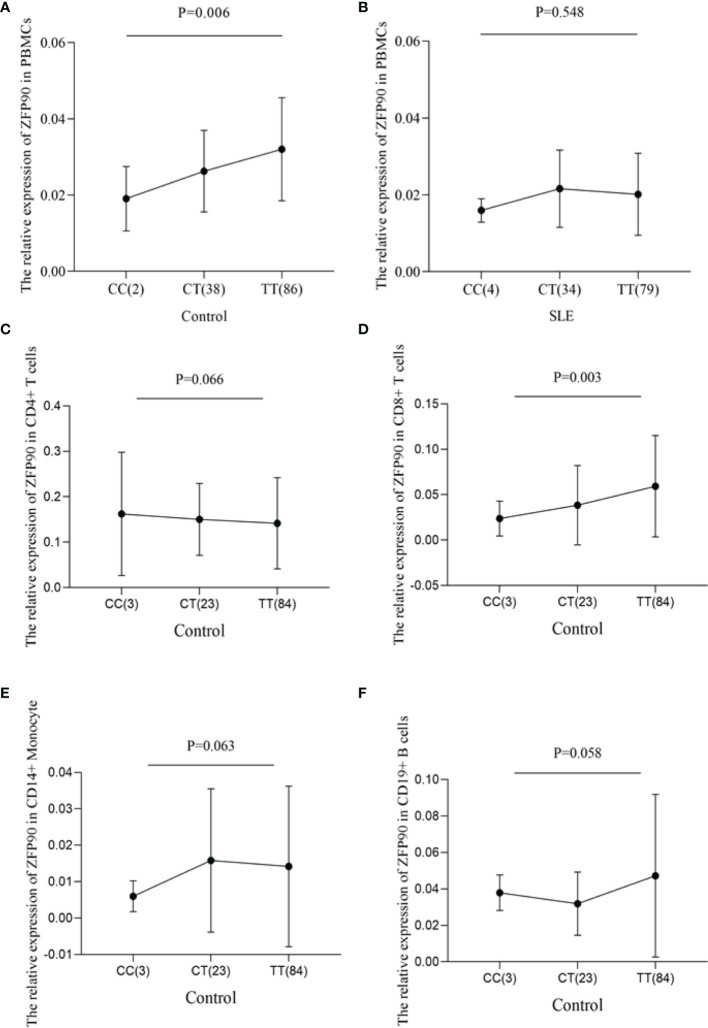
**(A)** The effect of rs1170426 on *ZFP90* mRNA expression levels in PBMCs from healthy controls. Of the 126 controls, 2 individuals with CC, 38 with CT and 86 with TT were analyzed. The group with “CC” homozygous has the lowest expression levels (*P =* 0.006). **(B)** The effect of rs1170426 on *ZFP90* mRNA expression levels in PBMCs from SLE cases. Of the 117 cases, 4 individuals with CC, 34 with CT and 79 with TT were analyzed. The expression did not significantly correlate with genotype of rs1170426 (*P* = 0.548). **(C–F)** The effect of rs1170426 on *ZFP90* mRNA expression levels in CD4+ T cells, CD8+ T cells, CD19+ B cells, and CD14+ monocytes from other 110 healthy controls. Of the 110 controls, 3 individuals with CC, 23 with CT and 84 with TT were analyzed. *ZFP90* expression levels of samples with risk allele “C” of rs1170426 were significantly decreased in CD8+ T cells (*P* = 0.003).

**Figure 3:**
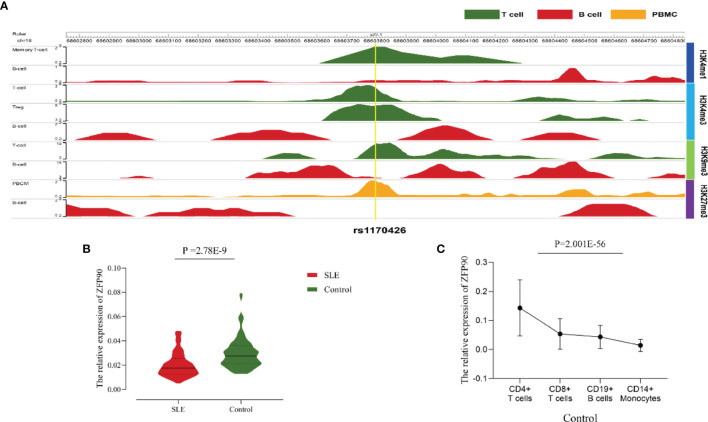
**(A)**The epigenome annotation results of rs1170426 in T cells (green), B cells (red) and PBMCs (orange). The signal can be found in T cells and PBMCs, while not in B cells. **(B)** Compared the *ZFP90* mRNA expression levels between SLE cases (n = 117, red) and healthy controls (n = 126, green) in PBMCs. The expression levels were lower in cases than in healthy controls (*P =*2.78E-9). **(C)** The *ZFP90* mRNA expression levels in four kinds of immune cells were remarkably different (*P* = 2.001E-56) and were higher in T cells.

**Figure 4:**
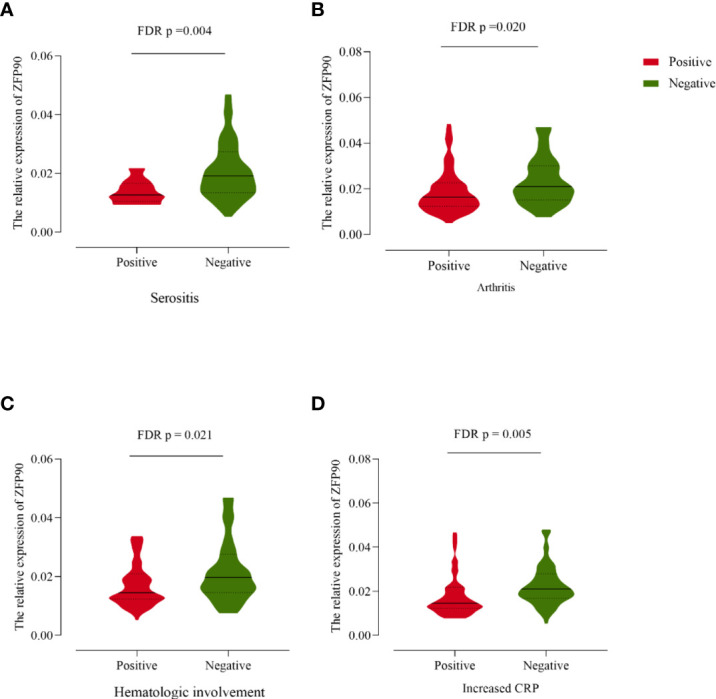
**(A)** The *ZFP90* mRNA expression levels were lower (FDR *p* =0.004) in cases with serositis than those without. **(B)** The levels were lower (FDR *p* = 0.020) in cases with arthritis than those without. **(C)** The levels were lower (FDR *p* = 0.021) in cases with hematologic involvement than those without. **(D)** The levels were lower (FDR *p* = 0.005) in cases with increased CRP than those without.

The authors apologize for these errors and state that this does not change the scientific conclusions of the article in any way. The original article has been updated.

## Publisher’s Note

All claims expressed in this article are solely those of the authors and do not necessarily represent those of their affiliated organizations, or those of the publisher, the editors and the reviewers. Any product that may be evaluated in this article, or claim that may be made by its manufacturer, is not guaranteed or endorsed by the publisher.

