# Supplementary material for: Assessing the Function of the ZFP90 Variant rs1170426 in SLE and the Association Between SLE Drug Target and Susceptibility Genes
Source: Front Immunol. 2021 Mar 16;12:611515. doi: 10.3389/fimmu.2021.611515 (PMC8008139; doi:10.3389/fimmu.2021.611515)

**Supplementary Figure 1.** The X-axis represents the time of EBV transfection of lymphocytes extracted from healthy controls, and the Y-axis represents the expression level of *ZFP90*. By using one-way analysis of variance and multiple test correction, the result shows FDR *p*=0.068.

**
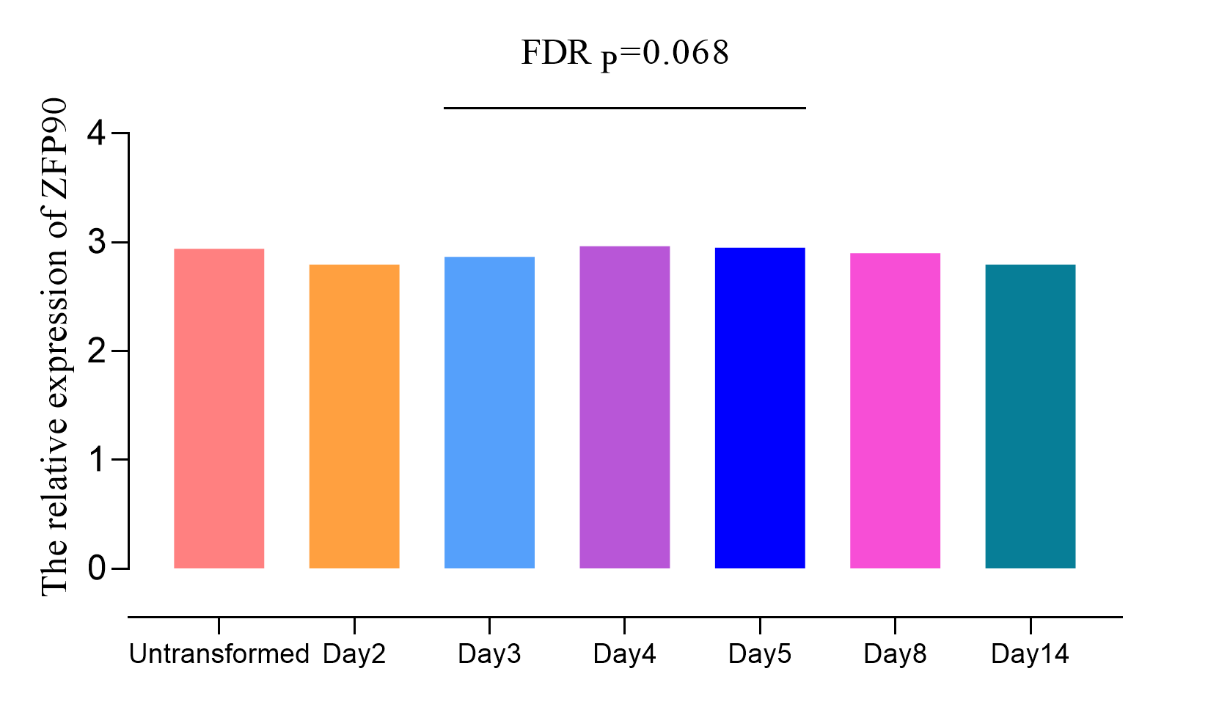
**

**Supplementary Figure 2.** (**A)** In the group of “CC” genotype**,** *ZFP90* mRNA expression levels was no significant difference in case-control (P= 0.004). (**B)** In the group of “CT” genotype, the expression levels were lower in SLE cases than in healthy controls (*P*= 0.004). **(C)** In the group of “TT” genotype**,** the expression levels were also lower in SLE cases than in healthy controls (*P*= 1.836E-9) .


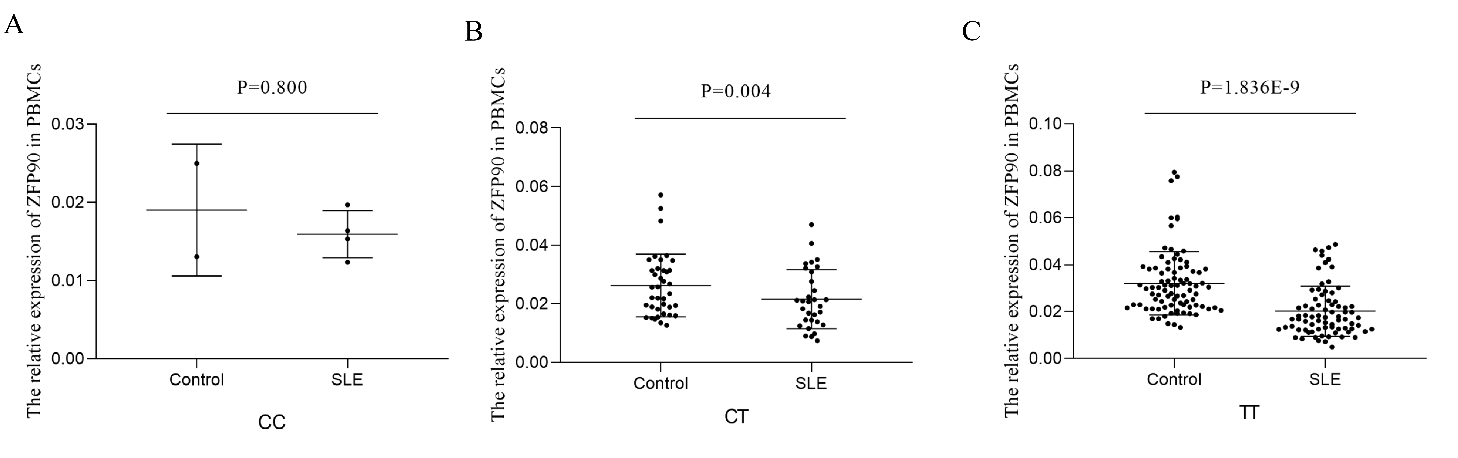

Supplement: Supplementary file 2 [file DataSheet_2.docx]
